# The effect of vitamin D and magnesium supplementation on clinical symptoms and serum inflammatory and oxidative stress markers in patients with COVID-19: a structured summary of a study protocol for a randomized controlled trial

**DOI:** 10.1186/s13063-023-07107-4

**Published:** 2023-02-06

**Authors:** Nahid Ramezani-Jolfaie, Ebrahim Eftekhar, Mohadese Dadinasab, Zahra Ghaeini Hesarooeyeh, Pooria Pakdaman, Farkhondeh Razmpour, Gholamali Javedan, Mahmood Khayatian, Mehdi Hassani Azad, Parivash Davoodian, Elham Brahimi, Shideh Rafati, Sara Nikoofal-Sahlabadi, Mohammad Mohammadi

**Affiliations:** 1 Food Health Research Center, Hormozgan University of Medical Sciences, Bandar Abbas, Iran; 2 Department of Community Medicine, School of Medicine, Hormozgan University of Medical Sciences, Bandar Abbas, Iran; 3 Endocrinology and Metabolism Research Center, Hormozgan University of Medical Sciences, Bandar Abbas, Iran; 4 Student Research Committee, School of Medicine, Hormozgan University of Medical Sciences, Bandar Abbas, Iran; 5 Molecular Medicine Research Center, Hormozgan Health Institute, Hormozgan University of Medical Sciences, Bandar Abbas, Iran; 6 Infectious and Tropical Diseases Research Center, Hormozgan Health Institute, Hormozgan University of Medical Sciences, Bandar Abbas, Iran; 7 Social Determinants in Health Promotion Research Center, Hormozgan Health Institute, Hormozgan University of Medical Sciences, Bandar Abbas, Iran; 8 Department of Pharmaceutics, Faculty of Pharmacy, Hormozgan University of Medical Sciences, Bandar Abbas, Iran

**Keywords:** COVID-19, Clinical trial protocol, Vitamin D, Magnesium, Dietary supplements

## Abstract

**Objectives:**

This study aims to evaluate the effect of vitamin D and magnesium supplementation on clinical symptoms and serum inflammatory and oxidative stress markers in patients with COVID-19.

**Trial design:**

This study is a 4-arm randomized, double-blind, placebo-controlled clinical trial with a factorial design and the intervention period is 3 weeks.

**Participants:**

This study is conducted on COVID-19 patients admitted to the Shahid Mohammadi hospital in Bandar Abbas, Iran, who are eligible for inclusion in the study. Patients are included only if they meet all of the following criteria: (1) aged from 18 to 65 years old; (2) confirmation of COVID-19 by RT-PCR test; (3) completing informed consent; (4) passing less than 48 h since the patient's hospitalization; (5) no skin or gastrointestinal allergies due to taking multivitamin supplements, vitamin D, and magnesium; and (6) having more than 30 breaths per minute and less than 93% oxygen saturation in room air and sea level. Patients are excluded if they have any of the following conditions: (1) pregnancy or lactation; (2) taking a daily multivitamin or take a vitamin D or magnesium supplement in the last month; (3) participating in other clinical trials; (4) renal failure or dialysis, severe liver disease or cirrhosis; (5) known diagnosis of hypercalcemia; (6) discharging from the hospital less than 24 h after the start of the intervention; (7) history of kidney stones in the last year; (8) transfer the patient to the ICU; (9) baseline vitamin D levels above 80 ng/ml; (10) baseline magnesium levels above 2.6 mg/dl; and (11) unwillingness of the patient to continue the study.

**Intervention and comparator:**

Participants will be randomly allocated to one of the four following groups: (A) vitamin D (two 50,000 IU capsules at the beginning of the study, two 50,000 IU capsules on the 4th day, one 50,000 IU capsule on the 11th day, and one 50,000 IU capsule on the 17th day) and magnesium supplement (300 mg/day); (B) vitamin D capsule and magnesium placebo; (C) magnesium supplement and vitamin D placebo; and (D) vitamin D placebo and magnesium placebo.

**Main outcomes:**

The resolution of clinical symptoms (fever, dry cough, shortness of breath, headache, myalgia, oxygen saturation, and mortality rate) and interpretation of laboratory assays (CRP, MDA, TAC, WBC, neutrophils count, lymphocytes count, ratio of neutrophils to lymphocytes, levels of 25 hydroxyvitamin D and magnesium) will be assessed in the study groups.

**Randomization:**

A computer-generated block randomization list is used for randomization.

**Blinding (masking):**

Investigators and patients are blinded to group allocation and treatment. A double-blind design is achieved using matched placebos.

**Numbers to be randomized (sample size):**

A total of 104 eligible patients are randomized into four groups of 26 subjects (1:1:1:1 allocation ratio).

**Discussion:**

With the rapid prevalence of COVID-19 in recent years, more attention has been paid to effective dietary supplementation to improve clinical symptoms and biochemical parameters in these patients. To our knowledge, this is the first study to evaluate the effects of vitamin D supplementation in combination with magnesium or alone with respect to this infectious disease. The findings of the current RCT will provide evidence regarding the effectiveness of dietary supplementation strategies to improve COVID-19 outcomes.

**Trial status:**

Ethical approval of the first version of the study protocol was obtained from the medical ethics committee of Hormozgan University of Medical Sciences, Bandar Abbas, Iran on May 30, 2021 (IR.HUMS.REC.1400.085). Currently, the recruitment phase is ongoing since August 23, 2021, and is anticipated to be complete by the end of August 2022.

**Trial registration:**

The study protocol was registered in the Iranian Registry of Clinical Trials (https://www.irct.ir; IRCT20210702051763N1) on August 14, 2021. https://www.irct.ir/trial/57413

**Full protocol:**

The full protocol is attached as an additional file, accessible from the Trials website (Additional file 1). In the interest in expediting dissemination of this material, the familiar formatting has been eliminated; this letter serves as a summary of the key elements of the full protocol.

**Supplementary Information:**

The online version contains supplementary material available at 10.1186/s13063-023-07107-4.

## Introduction

A new coronavirus (SARS-CoV-2) infection commonly known as COVID-19 disease has become an epidemic in all world regions since November 2019 beginning in the city of Wuhan, China. It has become even more prevalent in the rest of the world than inside China, and the epidemic is still spreading [1]. This infection can mostly lead to severe acute upper and lower respiratory and gastrointestinal tract symptoms, especially in older people or those with chronic underlying diseases such as diabetes, coronary artery disease, and hypertension [1]. COVID-19 involves different organs and causes many complications because of the reduced immune system function [2].

One of the most important issues of this new infectious disease is investigating and determining the possible effects of nutrition and dietary supplementation in the prevention and treatment of disease [3]. It should be mention that a growing body of evidence supported the nutritional components in immune system enhancement [4]. Vitamin D and magnesium are among the essential nutrients that play important roles in the physiological functions and immune system [5].

Vitamin D deficiency is a worldwide concern that has been shown to be associated with increased risk for several diseases and clinical conditions [6, 7]. The effectiveness of vitamin D in the stimulation of innate immunity and modulation of acquired immunity has been known [8]. A meta-analysis also concluded that vitamin D supplementation is safe and associated with protective effects against acute respiratory tract infection [9]. On the other hand, Meltzer et al. reported a higher COVID-19 risk in individuals with vitamin D deficiency who were not sufficiently treated (deficit group: 21.6% vs. sufficient group: 12.2%) [10]. It has been suggested that considering the favorable effects of treating vitamin D deficiency on respiratory tract infections, during this pandemic period, the circulating 25-OH vitamin D should maintain at optimal levels (75–125 nmol/L) through taking vitamin D supplements [11]. However, it is stated that there is still a need for more research work to better understand the role of vitamin D in reducing disease severity and mortality [11].

Magnesium is an important mineral in the activation of a variety of enzymes and physiological functions in metabolic regulation, vasomotor tone, and muscle contraction [12]. Its supplementation is supported to play roles in the prevention or treatment of various types of diseases related to the respiratory system [12]. It has been suggested that magnesium also contributes to the antioxidant and anti-inflammatory effects on lung injury [13]. Magnesium and vitamin D could have a positive effect on inflammation and reduce cytokine production and coagulation cascade [14]. Subclinical magnesium deficiency has been reported to be associated with the debilitation of the immune system [14, 15]. Indeed, since the intracellular free magnesium regulates the cytotoxic functions of natural killer and cytotoxic T cells, the reduced intracellular free magnesium levels can lead to defective expression of programmed cell death in these immune cells [16].

In a cohort study among hospitalized patients with COVID-19 older than 50 years, supplementation with 1000 IU/day vitamin D, 150 mg/day magnesium, and 500 mcg/day vitamin B_12_ simultaneously was associated with less need for oxygen therapy and ventilation. Interestingly, those patients who did not receive this supplementation needed a 3.5-fold higher rate of oxygen therapy throughout hospitalization versus those who received the vitamin/mineral supplement (61.5% vs. 17.6%, *P* = 0.006) [17].

Considering that vitamin D and magnesium balance are required for optimal biological function, it is likely that vitamin D and magnesium have synergistic effects in COVID-19. To our knowledge, there is no previous clinical trial of vitamin D supplementation in combination with magnesium or alone with respect to this infectious disease. Therefore, we aim to conduct a randomized, double-blind, placebo-controlled clinical trial with a factorial design to evaluate the effect of vitamin D and magnesium supplementation on clinical symptoms, inflammatory markers, and oxidative stress in patients with COVID-19.

## Materials and methods

### Trial design

This study is a 4-arm randomized (1:1:1:1 allocation ratio), double-blind, placebo-controlled clinical trial with a factorial design and the intervention period is 3 weeks. This study aims to evaluate the effect of vitamin D and magnesium supplementation on clinical symptoms and serum inflammatory and oxidative stress markers in patients with COVID-19 admitted to the Shahid Mohammadi hospital in Bandar Abbas, Iran.

### Participants

Inclusion criteria:

- Age from 18 to 65 years old
- Confirmation of COVID-19 by RT-PCR test
- Completing informed consent
- Passing less than 48 h since the patient’s hospitalization
- No skin or gastrointestinal allergies due to taking multivitamin supplements, vitamin D, and magnesium
- Having more than 30 breaths per minute and less than 93% oxygen saturation in room air and sea level

Exclusion criteria:

- Pregnancy or lactation
- Taking a daily multivitamin or take a vitamin D or magnesium supplement in the last month
- Participating in other clinical trials
- Renal failure or dialysis, severe liver disease or cirrhosis
- Known diagnosis of hypercalcemia
- Discharging from the hospital less than 24 h after the start of the intervention
- History of kidney stones in the last year
- Transfer the patient to the ICU
- Baseline vitamin D levels above 80 ng/ml
- Baseline magnesium levels above 2.6 mg/dl
- Unwillingness of the patient to continue the study

Patients should not participate in another study at the same time but the routine treatment of COVID-19 in all groups is continued.

### Intervention and comparator

Participants will be randomly allocated to one of the four following groups: (A) vitamin D (two 50,000 IU capsules at the beginning of the study, two 50,000 IU capsules on the 4th day, one 50,000 IU capsule on the 11th day, and one 50,000 IU capsule on the 17th day) and magnesium supplement (300 mg/day); (B) vitamin D capsule and magnesium placebo; (C) magnesium supplement and vitamin D placebo; and (D) vitamin D placebo and magnesium placebo.

To ensure patient compliance with the assigned treatment, the subjects are monitored weekly by telephone and, if necessary, in-person visits. The compliance rate will be calculated according to the number of delivered and returned capsules by the following formula: number of capsules delivered − number of capsules returned/number of capsules delivered.

The criteria for discontinuation are any medical complications during supplementation or at the request of the patient. However, there is no possibility of harm to participants due to the type of dietary intervention. If the assigned intervention is discontinued for any reason, the patient has to be excluded from the study.

### Data collection

At baseline, general characteristics of patients including age, gender, medications used, and underlying chronic diseases are collected by investigators using a paper-based record system. All outcomes of interest (clinical symptoms and laboratory markers) are assessed at baseline and at the end of the intervention period (3 weeks later).

Data entry will be done by one of the blind research team members and a double check is performed by another blinded team member. Written informed consent is collected for each patient at the beginning of the study by investigators. Dietary supplements and biochemical tests are provided free of charge for participants to increase their willingness to participate in the study. Data protection was done by keeping the identity of the patients anonymous by excluding their names throughout the study.

After an overnight fast (10–12 h), 10 ml venous blood samples are taken from patients in the morning. First, 4 ml blood is collected in ETDA tube (1 mg/ml) and used for complete blood cell (CBC) count. The remaining 6 ml blood is allowed to clot for 45 min at room temperature; serum is then isolated by centrifugation at 3000 rpm for 10 min and stored at – 70 °C until analysis. Blood samples are collected and assayed by lab investigators that are blinded to the patient assignments.

### Main outcomes

The resolution of clinical symptoms and interpretation of laboratory assays will be assessed in the study groups.

Clinical symptoms:

- Fever
- Dry cough
- Shortness of breath
- Headache
- Myalgia
- Oxygen saturation
- Mortality rate

Laboratory markers:

- C-reactive protein (CRP)
- Malondialdehyde (MDA)
- Total antioxidant capacity (TAC)
- White blood cell (WBC)
- Neutrophils count
- Lymphocytes count
- The ratio of neutrophils to lymphocytes
- Levels of 25 hydroxyvitamin D and magnesium

### Randomization

A computer-generated block randomization list is used for randomization. In this way, 24 blocks of 4 are made using codes A, B, C, and D, numbering from 1 to 24 (ABCD, ACBD, ABDC, ADBC, ACDB, ADCB, BACD, BCAD, BADC, BDAC, BCDA, BDCA, CABD, CBAD, CADB, CDAB, CBDA, CDBA, DABC, DBAC, DACB, DCAB, DBCA, DCBA) and then using R software, twenty-six blocks from blocks 1 to 24 are randomly selected as follows (3, 4, 7, 1, 2, 23, 11, 14, 18, 19, 6, 10, 6, 21, 17, 7, 9, 5, 22, 12, 20, 16, 13, 15, 3, 8). Allocated codes are then kept in sealed opaque envelopes, such that the numbers 1 to 104 are written on the envelopes and the codes A, B, C, and D are placed inside the envelopes as mentioned above order. For example, since block number 3 is the first choice, based on this block, the first person will receive treatment A, the second B, the third D, and the last will receive treatment C. This continues in the same way until 4 treatments are assigned to all patients.

### Blinding (masking)

Investigators and patients are blinded to group allocation and treatment. A double-blind design is achieved using matched placebos. Vitamin D and magnesium supplements and placebos are placed in the same package with codes of A, B, C, and D by a responsible person who is not aware of the study objectives. The code information will be given to the investigators after having done the statistical analysis. Moreover, to blind the participants, they are explained at the beginning of the study who will receive one of four types of intervention and will not be told the exact type of supplement.

Vitamin D supplements and placebos were provided by Zahravi Pharmaceutical Company (Iran). Magnesium placebos were made from starch with the same color and shape as magnesium supplements by the Faculty of Pharmacy of Hormozgan University of Medical Sciences.

### Sample size

A total of 104 COVID-19 patients aged 18 to 65 admitted to the Shahid Mohammadi hospital in Bandar Abbas, Iran, who are eligible for the study are recruited. They are randomly divided into four groups of 26 subjects.

Based on the findings of the previous study [18], the sample size was calculated by the G*Power software, where the effect size for MDA variable was 0.68, alpha error probability was 0.05, power = 0.8, number of groups = 4, number of measurements = 2, and the assumed correlation among the repeated measures was 0.5. Then, taking into account the dropout rate of 0.1, the sample size in each group was estimated to be 26 patients.

### Statistical analysis

The Kolmogorov–Simonov test will be used to check the normality of variable distribution. One-way ANOVA and Bonferroni post hoc tests will be used to analyze the differences between groups.

The mixed linear model will be used to estimate the treatment effect and the possible confounding variables will be adjusted in the model. IBM SPSS version 22 will be used for statistical analysis. *P*-value ≤ 0.05 will be considered a significant level in all statistical tests. To handle protocol non-adherence, the intention-to-treat (ITT) principle will be used to analyze data based on the original treatment assignment of the patients. Variables will be expressed as mean ± standard error or 95% confidence interval. The research team will not conduct interim analyses to stop or extend the trial because of the low-risk nature of the nutritional intervention in the present study. Indeed, early detection of the study hypotheses is unlikely and early termination of the intervention is not supposed to have any additional protection for patients.

## Discussion

With the rapid prevalence of COVID-19 in recent years, more attention has been paid to effective dietary supplementation to improve clinical symptoms and biochemical parameters in these patients [19, 20]. To our knowledge, this is the first study to evaluate the effects of vitamin D supplementation in combination with magnesium or alone with respect to this infectious disease. The findings of the current RCT will provide evidence regarding the effectiveness of dietary supplementation strategies to improve COVID-19 outcomes.

## Auditing trial conduct

The Deputy of Research of Hormozgan University of Medical Sciences has appointed an independent person who is not a member of the research team to monitor the research's safety, scientific validity, and integrity from the initial setup to final reporting. For this purpose, a study progress report must be sent every 6 months, and after the monitor’s verification, the budget will be paid to continue the trial.

Protocol amendments will be communicated to all study investigators, coordinators, and trial sponsors. If any modifications are needed, these will also be updated in the trial registry (IRCT).

## Trial status

Ethical approval of the first version of the study protocol was obtained from the medical ethics committee of Hormozgan University of Medical Sciences, Bandar Abbas, Iran on May 30, 2021 (IR.HUMS.REC.1400.085). Currently, the recruitment phase is ongoing since August 23, 2021 and is anticipated to be complete by the end of August 2022.

## Trial registration

The study protocol was registered in the Iranian Registry of Clinical Trials (https://www.irct.ir; IRCT20210702051763N1) on August 14, 2021. https://www.irct.ir/trial/57413

## Supplementary Information

**Additional file 1.** Reporting checklist for protocol of a clinical trial. Based on the SPIRIT guidelines.

## Data Availability

The final dataset of the trial will be available on request from the corresponding author via mohammadi.nut@gmail.com, after obtaining the permission of the medical ethics committee.
